# The Relationship between Oral and Dental Health Self-care and Hemoglobin A1c in Adults with Diabetes

**DOI:** 10.30476/DENTJODS.2021.87966.1300

**Published:** 2022-03

**Authors:** Atousa Haghdoost, Soheila Bakhshandeh, Zahra Ghorbani, Mahshid Namdari

**Affiliations:** 1 Dental Research Center, Research Institute of Dental Sciences, School of Dentistry, Shahid Beheshti University of Medical Sciences, Tehran, Iran; 2 Dept. of Community Oral Health, School of Dentistry, Shahid Beheshti University of Medical Sciences, Tehran, Iran; 3 Dept. of Biostatistics, School of Allied Medical Sciences, Shahid Beheshti University of Medical Sciences, Tehran, Iran

**Keywords:** Oral Health, Self-Care, Diabetes, Hemoglobin A1c

## Abstract

**Statement of the Problem:**

Due to the mutual relationship between periodontal diseases and diabetes, it seems that adopting oral self-care in a way to prevent and control the progress of periodontal diseases, improves the oral health of diabetic patients as well as their general health.

**Purpose:**

The aim of this study was to investigate the relationship between the oral self-care behaviors and the hemoglobin A1c (Hb A1c) levels in adults with diabetes.

**Materials and Method:**

In this cross-sectional study with convenience sampling, 120 adults between 18 to 50 years old, who had at least two healthy functional teeth, were selected from private endocrinology offices in Tehran in August 2019. The exclusion criteria were illiterate individuals and pregnant women. A standard questionnaire was used which included the information about demographic, diabetes, and self-care behaviors. The outcome variable was the latest Hb A1c rate.

**Results:**

The mean age of participants was 35.8±10.5 years. The average Hb A1c was 7.4± 1.55%. 35.0% of participants brushed their teeth twice a day or more and 60.8% flossed rarely. The proportion of Hb A1c <7% was higher in three groups including the participants who had information about the effect of periodontal disease on diabetes (*p*= 0.032), participants who brushed twice a day or more (*p*= 0 .014), and those who used dental floss once a day or more (*p*< 0.001). The likelihood of having Hb A1c <7% in participants who had information about the effect of periodontal disease on diabetes was about three times more than those who had no information (OR= 3.05, *p*= 0.036). Furthermore, it was about six times higher in participants who used dental floss once a day or more than those who used rarely (OR= 5.66, *p*= 0.001).

**Conclusion:**

Results of the present study show that people who had better oral health self-care behaviors had better Hb A1c and diabetes control.

## Introduction

Diabetes is one of the most common metabolic diseases in the world and its prevalence in adults has increased in the last decade [ 1 - 2 ]. The International Diabetes Federation estimated that the number of adults with diabetes would reach 592 million, by 2035 [ 3 ]. It is estimated that by 2030, there will be 9.2 million people with diabetes in Iran [ 4 ]. 

One criterion for diagnosis and controlling diabetes, as well as preventing its complications, is measuring the level of glycosylated hemoglobin A1c (Hb A1c) [ 5 - 6 ]. This test is one of the criteria for estimating the severity of diabetes complications [ 5 - 6 ]. Its concentration indicates the average level of blood sugar over the past three months [ 5 - 6 ]. Evidence shows that Hb A1c<7% can prevent microvascular complications. Diagnosis criterion for diabetes is Hb A1c >6.5% [ 5 - 7 ]. 

The most important complications of diabetes include its effects on vascular tissues and organs such as kidneys, retina, and neurons. Similar changes in small blood vessels can occur in the oral cavity tissue [ 8 ]. Oral disorders such as dry mouth, sialosis, taste disturbance, oral lichen planus and lichenoid reaction, gingival and periodontal diseases, dental caries, tongue disorder and oral candidiasis are common among diabetic patients [ 9 ]. For example, periodontitis, an inflammation of the gingiva and tooth supporting structures, is the most common oral disease in people with diabetes. The severity of periodontitis increases with poor control of diabetes [ 10 - 11 ]. According to literatures people with diabetes with Hb A1c>9% had a significantly higher prevalence of periodontitis compared to non-diabetics [ 12 - 13 ]. Uncontrolled diabetes is a risk factor for periodontal disease and treatment of periodontal disease improves diabetes control [ 14 - 15 ].

Given the strong evidence for mutual relationship between diabetes and increasing the risk of periodontal disease, it seems that oral health self-care behaviors such as brushing, flossing, and regular dental visits could improve the oral and general health of diabetic patients. Therefore, today the attention of health care professionals is focused on this issue [ 16 - 18 ]. Since the people with diabetes are less aware of the mutual relationship between diabetes and periodontal disease, researchers believe that more education should be given to people with diabetes [ 19 - 23 ].

Moreover, it is supposed that risk factors such as poor diet, smoking, stress, and so on are common issues in many chronic diseases such as diabetes and oral diseases [ 24 ]. Considering the beneficial effects of oral health self-care behaviors on diabetes control, the aim of this study was to investigate the relationship between oral health self-care behaviors and Hb A1c levels in adults with diabetes.

## Materials and Method

This cross-sectional study has been registered in the Ethics Committee of Shahid Beheshti University of Medical Sciences (ID: IR.SBMU.DRC.REC.1398.014). The present study with convenience sampling was conducted among adults with diabetes in Tehran, in August 2019. Adults between 18 to 50 years old, who had at least two healthy functional teeth, were included in this study. One hundred and twenty individuals who referred to three private endocrinology offices were selected in different areas of Tehran. Illiterate individuals and pregnant women were excluded from the study. The researcher clearly stated the study objectives and assured the participants of the confidentiality of the information and then the informed consents were obtained.

A standard questionnaire [ 25 ] consisting of three sections was administered by the researcher. The first part contained demographic information (age, gender and level of education), the second part contained information about diabetes (type of diabetes, duration of diabetes, the latest Hb A1c rate), and the third part consisted the questions concerning the oral self-care behaviors (brushing, flossing, regular dental visits). After filling out the questionnaire by the participants and delivering it to the researcher, they were referred to their endocrinologist for a visit. 

The outcome variable was the latest Hb A1c rate which was divided into two groups of <7% and >7%. The explanatory variables included a yes/no question regarding regular dental visit: ("What was the main reason for your last dental visit?" with these options provided: "Frequent and regular examinations (yes); Pain or other emergencies/Continuation of previous treatment/I do not remember (no)". The two other questions were related to brushing (less than twice a day/twice a day and more) and flossing (rarely/once a day and more). Questions about age (18-30 years, 31-40 years, 41-50 years), level of education (university/non-university), type of diabetes (type one/type two), duration of diabetes (under 7 years, 7-12 years, and over 12 years), smoking (yes/ no), extracted teeth (yes/no) and information about the effect of periodontal disease on diabetes (yes/no) were considered as correlation variables. Then all of data were collected from the questionnaires. 

The SPSS software, version 21 was used to analyze the data. The descriptive statistic indices such as mean and standard deviation were extracted. Bivariate statistical analysis was performed by independent t-test, Mann-Whitney, and Chi-Square tests. The binary logistic regression test was used to evaluate the relationship between Hb A1c and explanatory variables with a significance level of less than 0.05. In regression, adjusted models were used. In model one, gender, and age group were evaluated. In model two, gender, age group, and level of education were evaluated. The type of diabetes and duration of diabetes were added to model three. The oral health self-care behaviors (smoking, tooth extraction, regular dental visit, information about the relationship between periodontal disease and diabetes, brushing and flossing) were added to model four.

## Results

Of 120 participants with a mean age of 35.8±10.5, 63 were female (52.5%) and 57 were male (47.5%). Of the total study population, 55.0% had type 1 diabetes and 45.0% had type 2 diabetes. The average duration of diabetes was reported 11.2±7.45 years. The mean of the latest Hb A1c rate was 7.4±1.55% (Range=4.9% to 13.2%). 

Of the study population, 90 (75.0%) had never smoked. 48.3% visited a dentist less than a year ago; 40.0% reported pain or other emergencies as the main reason for their last dental visit. Among those who did not visit the dentist for more than two years, 20.0% reported that they did not have a dental problem and 18.3% were busy and did not have time. The most common treatment reported in the last dental visit was endodontic (32.5%) and caries restoration (27.5%). The average number of permanently extracted teeth was 1.8±2.90. 22.5% reported regular dental visits. Among 120 people, 78 (65.0%) brushed their teeth less than twice a day and 42 (35.0%) brushed twice a day and more. In addition, 73 people (60.8%) used dental floss rarely and 47 people (39.2%) flossed once a day and more. In general, only 8.3% of people were referred to a dentist on the advice of their diabetes specialist. Among the study population, only 30.0% had information about the effect of gum disease on diabetes (Table 1). 

**Table 1:** Characteristics of the participants (n=120)

Variables			N	%
Demographic information	Gender	Female	63	52.5
		Male	57	47.5
	Age	18-30 years	39	32.5
		31-40 years	39	32.5
		41-50 years	42	35.0
	Level of education	University	62	51.7
		Non-university	58	48.3
Information about diabetes	Type of diabetes	Type one	66	55.0
		Type two	54	45.0
	Duration of diabetes	Under 7 years	41	34.2
		7-12 years	30	25.0
		Over 12 years	49	40.8
	Latest Hb A1c^a^ rate	<7%	66	55.0
		>7%	51	42.5
		Without response	3	2.5
Self-care behaviors	Regular dental visit	Yes	27	22.5
		No	92	76.7
		Without response	1	0.8
	Brushing	Less than twice a day	78	65.0
		Twice a day and more	42	35.0
	Flossing	Rarely	73	60.8
		Once a day and more	47	39.2

a Hemoglobin A1c

People who had information about the effect of periodontal disease on diabetes had the highest Hb A1c <7% (*p*= 0.032). The most recent Hb A1c <7% was higher in people who brushed their teeth twice a day or more, as well as those who used dental floss once a day or more (*p*< 0.001 and *p*= 0.014 respectively) (Table 2).

**Table 2:** The relationship between Hb A1c^a^ and information about demographic, diabetes and self-care behaviors

Variables			Latest Hb A1c^a^ rate				
			<7% (n=66)		>7% (n=51)		*p*
			N	%	N	%	
Demographic information	Gender	Female	37	61.7	23	38.3	0.239
		Male	29	50.9	28	49.1	
	Age	18-30 years	22	56.4	17	43.6	0.239
		31-40 years	24	66.7	12	33.3	
		41-50 years	20	47.6	22	52.4	
	Level of education	University	38	63.3	22	36.7	0.121
		Non-university	28	49.1	29	50.9	
Information about diabetes	Type of diabetes	Type one	40	61.5	25	38.5	0.211
		Type two	26	50.0	26	50.0	
	Duration of diabetes	Under 7 years	21	52.5	19	47.5	0.828
		7-12 years	17	58.6	12	41.4	
		Over 12 years	28	58.3	20	41.7	
Self-care behaviors	Smoking	Yes	17	56.7	13	43.3	0.974
		No	49	56.3	38	43.7	
	extracted teeth	Yes	37	54.4	31	33.3	0.608
		No	29	53.9	20	40.8	
	Regular dental visit	Yes	18	66.7	9	40.9	0.242
		No	48	53.8	41	46.1	
	Information about the effect of gum disease on diabetes	Yes	25	71.4	10	28.6	0.032
		No	41	50.0	41	50.0	
	Brushing	Less than twice a day	36	48.0	39	52.0	0.014
		Twice a day and more	30	71.4	12	28.6	
	Flossing	Rarely	31	42.5	42	57.5	0.000
		Once a day and more	35	79.5	9	20.5	

a Hemoglobin A1c

The results of logistic regression analysis in the final model showed that the likelihood of having Hb A1c <7% in participants who had information about the effect of periodontal disease on diabetes was about three times more than those who had no information (OR= 3.05, p= 0.036). Furthermore it was about six times higher in participants who used dental floss once a day or more compared to those who rarely flossed (OR= 5.66, *p*= 0.001) (Table 3).

**Table 3:** Association between information about demographic, diabetes and self-care behaviors with latest Hb A1c^a^ rate

Variables			Model 1 OR(95%CI)	Model 2 OR(95%CI)	Model 3 OR(95%CI)	Model 4 OR(95%CI)
Latest Hb A1c^a^ rate	Gender	Male	1	1	1	1
		Female	1.57(0.75;3.31)	1.68(0.79;3.59)	1.58(0.73;3.44)	2.08(0.77;5.64)
	Age	41-50 years	1	1	1	1
		31-40 years	2.22(0.88;5.61)	2.14(0.84;5.45)	2.04(0.75;5.56)	2.19(0.76;6.28)
		18-30 years	1.40(0.58;3.39)	1.45(0.59;3.54)	1.06(0.32;3.51)	1.68(0.53;5.30)
	Level of education	Non-university		1	1	1
		University		1.85(0.86;3.95)	1.88(0.86;4.09)	1.46(0.59;3.59)
	Type of diabetes	Type two			1	
		Type one			1.48(0.45;4.81)	
	Duration of diabetes	Under 7 years			1	
		7-12 years			1.47(0.50;4.29)	
		Over 12 years			1.10(0.38;3.16)	
	Smoking	No				1
		Yes				2.07(0.68; 6.29)
	Extracted teeth	No				1
		Yes				1.06(0.42; 2.70)
	Regular dental visit	Yes				1
		No				1.09(0.35; 3.41)
	Information about the effect of gum disease on diabetes	No				1
		Yes				3.05(1.08;8.62)
	Brushing	Less than twice a day				1
		Twice a day and more				1.06(0.37;2.99)
	Flossing	Rarely				1
		Once a day and more				5.66(2.08;15.37)

a Hemoglobin A1c

OR, odds ratio; 95% CI, confidence interval.

Model 1: adjusted for gender and age.

Model 2: adjusted for gender, age and level of education.

Model 3: adjusted for gender, age, level of education, type of diabetes and duration of diabetes.

Model 4: adjusted for gender, age, level of education, smoking, extracted teeth, regular dental visit, information about the effect of gum disease on diabetes, brushing and flossing.

Bold: *p* < 0.05

## Discussion

The results of the study showed that the most recent Hb A1c was better in participants having information about the effect of periodontal disease on diabetes, those who brushed twice a day or more, and those who used dental floss on a daily basis. It seems that being aware of the effect of periodontal disease on diabetes and paying more attention to oral health self-care behaviors such as brushing and flossing could improve both the oral health and diabetes control. This outcome is in accordance with the results yielded by the studies of Farahat *et al*. [ 26 ] and Merchant *et al*. [ 27 ].

In line with previous studies [ 28 - 29 ], the present study showed that half of the people with diabetes had visited a dentist within the past 12 months. However, a study from Saudi Arabia reported that only 12.6% of people had this pattern of visit [ 19 ].

In the study of Shanmukappa *et al*. [ 30 ], the main reason for the last dental visit was regular visits. In contrast, in this study and in the study of Aggarwal *et al*. [ 31 ], the main reason for the last dental visit was pain. 

According to the present study, the most common reasons for not having dental visits for more than two years were reported not having a dental problem, being busy, and not having enough time, respectively. In the study of Aggarwal *et al*. [ 31 ], the most common reasons for not having dental visits for more than two years were lack of knowledge about the need for regular dental visits and not having a dental problem. 

The results of the current study showed an improvement in the brushing habit of individuals compared to previous studies in Iran [ 32 ] and other countries [ 19 - 20 , 30 , 33 ] that may be due to the increased information about oral health in today's advanced world compared to the past. Furthermore, according to the results of the current study, two-thirds of participants rarely used dental floss, which is not much different from previous studies [ 19 , 21 ]. 

Comparing our results with the results of previous studies, more participants used to go to a dentist on the advice of their diabetes specialist in the past. This may indicate that our endocrinologists do not pay attention to the importance of oral health in diabetic patients [ 30 - 31 ]. 

In the present study and the study of Shanmukappa *et al*. [ 30 ], one-third of the study population had information about the effect of periodontal disease on diabetes, while in the study of Shimpi *et al*. [ 29 ], more than the half of study population had information about the effect of periodontal disease on diabetes. The authors assume that this might be due to the fact that in developing countries, people with diabetes also suffer from other health-related issues, and also are not aware of the effect of periodontal disease on diabetes.

One of the limitations of the current study was the impossibility of random sampling, so our study population cannot be representative of the population with diabetes in Tehran. In addition, according to the study in a private office in Tehran, it is better to conduct further studies in hospitals and public health centers in several different cities. Furthermore, due to the use of questionnaire as the only method of data collection, a number of questions have not been answered. Using the interview method to collect data and obtaining some information from patients' records are recommended for future studies. In addition, there are some other factors, affecting the evaluated relationships that have not mentioned in this study including insurance system, economic factors, and so on which are suggested to be elucidated in future studies.

## Conclusion

The results of the present study showed that the most recent Hb A1c was better in participants having information about the effect of periodontal disease on diabetes, those who brushed twice a day or more, and those who used dental floss on a daily basis. It should be considered that health care personnel have a duty to encourage patients to practice better oral self-care, as a part of diabetes self-care counseling.

## Acknowledgement

Thanks to Dr. Rajab and Dr. Kalbasi from the Iranian Diabetes Society for their kind help to this research project. This study was funded by Research Affairs of Dental School, Shahid Beheshti University of Medical Sciences, Tehran, Iran.

## Conflict of Interest

None declared.
